# Estrogen regulates PDPK1 to promote cell proliferation in epithelial ovarian cancer

**DOI:** 10.1016/j.heliyon.2024.e40296

**Published:** 2024-11-08

**Authors:** Yajie Wang, Huanchao Chang, Xiuwen Li, Hairong Zhang, Qianqian Zhou, Shengjian Tang, Di Wang

**Affiliations:** a School of Basic Medical Sciences, Shandong Second Medical University, Weifang, Shandong 261053, China; b Plastic Surgery Institute, Shandong Second Medical University, Weifang, Shandong 261053, China; c Affiliated Hospital of Shandong Second Medical University, Weifang, Shandong, China

**Keywords:** EOC, PDPK1, Estrogen, ESR1, Oncogene

## Abstract

Epithelial ovarian cancer (EOC) is a common estrogen-sensitive tumor that poses a serious threat to women 's health, and the mortality rate of EOC ranks first among malignant tumors in females. Studies have indicated a strong link between estrogen abnormality and EOC progression. We accidently found that 3-phosphoinositide-dependent protein kinase-1 (PDPK1) is highly expressed in EOC tissues. Further, estrogen also up-regulates the expression of PDPK1 in EOC cells. Notably, the expression of PDPK1 is controlled strictly, and its expression can determine the fate of cells. However, to date, the molecular mechanism by which estrogen elicits PDPK1 expression in EOC cells, and the role of PDPK1 in estrogen-driven EOC cells are not well defined. In this research, we found that a high expression of PDPK1 was associated with poor prognosis in patients with ovarian cancer. Further, estrogen stimulated the increase of PDPK1 protein expression through estrogen receptor ESR1. The depletion or overexpression of PDPK1 affected the inhibition or amplification of estrogen-driven EOC cell proliferation, and the knockdown of PDPK1 suppressed the migration of EOC cells by estrogen while promoting cell apoptosis. This suggests a critical functional association between estrogen and PDPK1 in the process of EOC. The expression of messenger RNA for cyclin A1, cyclin-dependent kinase 2 (CDK2), matrix metallopeptidase 2 (MMP2), and bcl-2 associated x protein (Bax) is regulated by PDPK1 under estrogen treatment. Our results indicated that PDPK1 plays a role as an oncogene in the development of EOC; hence, elucidating the mechanism by which estrogen promotes EOC progression by regulating PDPK1 expression.

## 1 Introduction

Epithelial ovarian cancer (EOC) is a leading cause of cancer-related death in women worldwide, with a mortality rate of about 150,000 cases per year [[1], [2], [3]]. Based on the histopathological, immunohistochemical, and molecular genetics analysis, EOC can be categorized as at least five clinicopathological types as follows: high-grade serous carcinoma (HGSC), endometrioid carcinoma (EC), clear cell carcinoma (CCC), mucous carcinoma (MC), and low-grade serous carcinoma (LGSC) [[4], [5], [6], [7], [8]]. Among these, serous ovarian cancer makes up approximately 85 % of all types of EOC. Due to multiple pathological types of EOC, using population-based screening has not been effective. This indicates an urgent need to explore the molecular regulation mechanism of EOC development to achieve early diagnosis of patients and screen promising therapeutic targets. Although the fact that the molecular characteristics of EOC remain elusive, sustained imbalance of estrogen levels, which is regarded as the principal element in the progression and metastasis of EOC [9]. Estrogen, a steroid product of the ovary, plays a crucial part in the development and maturation of various organs [10]. And the role of estrogen is mainly mediated by estrogen receptors (ERs) for the expression regulation of downstream target genes [11,12]. At present, a series of studies have focused on the mechanism of estrogen-driven EOC, but the molecular mechanism by which estrogen regulates the behavior of EOC involves complex signaling pathways and its potential gene targets are not fully explicit.

3-phosphoinositide-dependent kinase 1(PDPK1), a serine/threonine protease, belongs to the AGC kinase family. It was originally discovered in 1997, due to its ability to phosphorylate Akt [13]. Additionally, PDPK1 is also responsible for the phosphorylation of many other kinases in the AGC kinase family, such as the members of protein kinase C (PKC) family [[14], [15], [16]]. Considering that the downstream effectors of PDPK1 are reportedly involved in pathological phenotypes such as cell replication, apoptotic escape, invasion, and propagation, so it is not surprising that its expression affects the development of the organism [17,18]. Furthermore, PDPK1 expression is strictly necessary for embryonic development and maintenance of signs in adulthood in mammals. And previous study demonstrated that the PDPK1 knockout mouse model died at E9.5 days, and phenotypic changes such as circulatory and nervous system defects were noted [19]. Many normal tissues show weak PDPK1 expression, but the opposite is true in a series of tumor tissues. In breast cancer, PDPK1 is highly expressed, which is associated with poor prognosis. Beyond expression data, artificially increasing PDPK1 expression in breast cancer cells promotes cells proliferation and invasion [[20], [21], [22]]. In addition, the upregulation of PDPK1 is frequently detected in prostate cancer, esophageal squamous cell cancer, gastric cancer, and acute myeloid leukemia [[23], [24], [25], [26]]. Recent studies have shown that PDPK1 is involved in the invasiveness of ovarian cancer via short Ron receptor tyrosine kinase and COL11A1 [27,28]. Of note, the pharmacological inhibition of PDPK1 has been found to increase the effect of chemotherapy drugs on ovarian cancer [29]. However, the expression of PDPK1 in EOC and its upstream regulatory mechanism remains unclear. Under these premises, our interest in PDPK1 as a target to prevent EOC progression is increasing.

In this study, we detected the role and hormonal regulation mechanism of PDPK1 in SK-OV-3 cells, which are considered a useful model to understand the growth, migration, and apoptosis of human EOC cells. PDPK1 shows showed high expression in both EOC tissues and SK-OV-3 cells. The treatment of SK-OV-3 cells with estrogen can induce PDPK1 expression via ESR1. Moreover, the depletion of PDPK1 significantly reversed estrogen-mediated enhancement of proliferation and migration and in turn overexpression of PDPK1 increased estrogen-mediated proliferation and migration. Collectively, our study provides novel insights regarding the role of estrogen in regulating the expression and function of PDPK1 in the development of EOC.

## 2 Methods

### 2.1 Tissues samples and immunohistochemical (IHC) staining

EOC tissues and benign ovarian tissues were obtained from the Department of Gynecology of the Affiliated Hospital of Shandong Second Medical University. The EOC tissues were collected post-diagnosis but before chemotherapy. The protocol of the study program was approved by the Shandong Second Medical University Ethics Committee (063/2018), and pathological diagnoses of ovarian samples were made by two specialized gynecological pathologists based on the World Health Organization (WHO) classification for ovarian cancer. The study was approved by all patients before the start of the study and in accordance with all regulations. After obtaining fresh tissues, the tissues were washed with normal saline, and fixed in 4 % paraformaldehyde (Servicebio, Wuhan, China) thereafter. On the next day, the fixed tissue was dehydrated, embedded, and finally, 7 μm-thick sections were made. Paraffin sections were dewaxed with xylene and rehydrated in a concentration gradient of ethanol solution. The rehydrated tissue sections were placed in a boiling water bath for 25 min with an antigen retrieval solution that contained citric acid, cooled with running water, and then transferred to 0.3 % hydrogen peroxide in methanol for 40 min. After blocking in 3 % BSA in PBS for 60 min, the tissue sections were incubated with antibody against PDPK1 at 4 °C overnight, washed with PBS, and incubated with goat anti-rabbit HRP IgG for 60 min at 37 °C. Finally, the nucleus was stained with DAPI (Solarbio, Beijing, China) and the slides were observed using fluorescence microscopy.

### 2.2 Cell culture

Human EOC cells (SK-OV-3) were purchased from Procell Life Science & Technology Co., Ltd. (Procell, Wuhan, China). They were cultured in McCoy'5a (Procell, Wuhan, China, PM150714) with 10 % FBS and 1 % penicillin‐streptomycin solution. Normal human ovarian epithelial cells (IOSE80) were obtained from Innovatbio Life Technology Co. Ltd. (Innovatbio, Wuxi, China). They were cultured in DMEM with 10 % FBS and 1 % penicillin‐streptomycin solution. All the cells were placed in an incubator, which was set to 37 °C at 5 % CO_2_.

### 2.3 TCGA data set

To explore the potential prognostic significance of PDPK1 in ovarian cancer as well as the correlation between PDPK1 expression and Ki-67 expression, we obtained transcriptional profiles and follow-up information regarding ovarian cancer patients by querying HPA, TIMER, and GEPIA databases.

### 2.4 Protein extraction and western blotting

Protein extraction and western blotting were performed after modifying previously described methods [30,31]. The uncropped and unadjusted versions of the western blotting images are presented in Fig. S1.

### 2.5 Plasmid DNA and siRNA treatment

The GFP-PDPK1 plasmid was constructed by inserting an open reading frame of the PDPK1 gene into the pEGFP-C1 plasmid vector. The SK-OV-3 cells were transfected with pEGFP-C1 or PDPK1-pEGFP-C1 at a density of 70–90 %, according to the Lipofectamine 3000′s protocol. Regarding siRNA transfections, the sequences of ESR1/ESR2/PDPK1 siRNA and NC siRNA are shown in Table 1. The siRNA transfections were performed at a density of 30–50 %. Western blotting was used to detect the efficiency of plasmid or siRNA transfection.

**Table 1:** SiRNA sequences for RNA interference in this experiment.

SiRNA name	(5′-3′) nucleotide sequence
NC	Sense UUC UCC GAA CGU GUC ACG UTT
	Antisense ACG UGA CAA GUU CGG AGA ATT
ESR1-homo-1019	Sense GGA GAA UGU UGA AAC ACA ATT
	Antisense UUG UGU UUC AAC AUU CUC CTT
ESR1-homo-1338	Sense GGA UUU GAC CCU CCA UGA UTT
	Antisense AUC AUG GAG GGU CAA AUC CTT
ESR1-homo-1947	Sense GGG CUC UAC UUC AUC GCA UTT
	Antisense AUG CGA UGA AGU AGA GCC CTT
ESR2-homo-1494	Sense GCU GAU GUG GCG CUC AAU UTT
	Antisense AAU UGA GCG CCA CAU CAG CTT
ESR2-homo-1891	Sense CAU CUG CUC AAC AUG AAG UTT
	Antisense ACU UCA UGU UGA GCA GAU GTT
ESR2-homo-1606	Sense CUC CUG GCA ACU ACU UCA ATT
	Antisense UUG AAG UAG UUG CCA GGA GTT
PDPK1-homo-651	Sense GCU GAG AUU GUG UCU GCU UUA TT
	Antisense UAA AGC AGA CAC AAU CUC AGC TT
PDPK1-homo-1322	Sense CAA CAU AGA GCA GUA CAU UCA TT
	Antisense UGA AUG UAC UGC UCU AUG UUG TT
PDPK1-homo-1644	Sense ACG CCU AAC AGG ACG UAU UAU TT
	Antisense AUA AUA CGU CCU GUU AGG CGU TT

### 2.6 Immunofluorescence assay

The cells were inoculated into a 24-well plate with slides at the bottom, and the cells were treated after 24 h. After a different treatment, 4 % paraformaldehyde was added to fix the cells, and the cells were washed with PBS after fixation. Thereafter, the cells were blocked with 3 % BSA (Solarbio, Beijing, China), and incubated with an antibody against PDPK1 overnight at 4 °C. On the next day, blocking was done with ABflo® 594-conjugated Goat Anti-Rabbit IgG antibody at room temperature for 1 h, and the nucleus was stained with DAPI (Solarbio, Beijing, China). Finally, the fluorescence effect was observed, and imaging was performed with a fluorescence microscope (Olympus, Japan).

### 2.7 Antibodies

All the antibodies were diluted using an antibody diluent (Absin, Shanghai, China). The anti-PDPK1, anti-ESR1, and anti-ESR2 antibodies were obtained from ABclonal (ABclonal, Wuhan, China). Further, ABflo® 594-conjugated Goat Anti-Rabbit IgG antibody (AS039,1:1000) and AP Goat Anti-Rabbit IgG antibody were also obtained from ABclonal. Estrogen (E2), and the ER inhibitor Fulvestrant was purchased from Abcam Company (Abcam, UK). The GPR30 inhibitor G15 was obtained from APxBIO Company (APxBIO, USA).

#### 2.7.1 Quantitative real-time PCR

For the SK-OV-3 cells, total RNA was extracted using the TRIzol buffer (Solarbio, Beijing, China) according to the manufacturer's protocol. The purity and concentration of the total RNA were measured using a microspectrophotometer (Thermo Fisher Scientific, USA). The total RNA was reverse transcribed into cDNA using the Bioteke super RT Kit (Bioteke, Beijing, China). Thereafter, qRT-PCR was performed. Pairs of primers designed as follows: GAPDH, forward: 5′-TGACATCAAGAAGGTGGTGAAGCAG-3′, reverse: 5′-GTGTCGCTGTTGAAGTCAGAGGAG-3′; Cyclin A1, forward: 5′- GAAGCAGCCAGACATCACGGAAG-3′, reverse: 5′- CCAGGAAGTTGACAGCCAGATACAG-3′; CDK2, forward: 5′-TGCCTGATTACAAGCCAAGTTTCCC-3′, reverse: 5′-TTGCGATAACAAGCTCCGTCCATC-3′; MMP-2, forward: 5′- CACCTACACCAAGAACTTCCGTCTG-3′, reverse: 5′- GTGCCAAGGTCAATGTCAGGAGAG-3′; Bax, forward: 5′- GATGCGTCCACCAAGAAGCTGAG-3′, reverse: 5′- CACGGCGGCAATCATCCTCTG -3′. The calculation method was as mentioned above [31,32].

### 2.8 Wound healing assay

For the wound healing assay, before seeding cells, a starting line was drawn in the middle of a well in a 6-well plate with a surgical blade and a straight ruler. SK-OV-3 cells, SK-OV-3 cells with PDPK1 deletion, and SK-OV-3 cells with GFP or PDPK1-GFP expression were seeded in a 6-well plate with straight line markings. The cells were cultured with a whole serum, at the density of cells reached of 80–90 %, a 1000 μL tip is used to scrape cells. After washing with PBS, the cells were cultured with McCoy's 5A serum-free medium. The plates were then visualized using an inverted microscope (Olympus, Japan).

### 2.9 EdU assay

For the Edu assay, SK-OV-3 cells, SK-OV-3 cells with PDPK1 deletion, and SK-OV-3 cells with GFP or PDPK1-GFP overexpression were seeded in 24-well plates with coverslips at the bottom of each plate. After 1 μM E2 stimulation for 24 h, the SK-OV-3 cells were exposed to 10 μM of EdU solution for 5 h at 37 °C and then fixed with 4 % paraformaldehyde solution in dark. After washing the cells with PBS, 0.5 % Triton X-100 was used to permeabilize the cells. The cells were stained using 5 μg/mL Hoechst 33342 solution according to the manufacturer's instructions. The plates were then visualized by a fluorescence microscope (Olympus, Japan).

### 2.10 Flow cytometry detection

SK-OV-3 cells and SK-OV-3 cells with PDPK1 deletion were treated with 1 μM E2 for 72 h. An Annexin V-FITC/PI apoptosis detection kit (BD Pharmingen, USA) was used to detect apoptosis of SK-OV-3 cells under different treatments according to the manufacturer's instructions. The results were observed using flow cytometry (BD FACSCalibur, USA).

### 2.11 Statistical analysis

All statistical analyses of the results were performed using SPSS20.0(SPSS Inc., Chicago,USA). The differences in protein expression levels, cell proliferation, cell migration and cell apoptosis between groups were analyzed using the Student's t-test. Correlation analysis was performed using Spearman test.

## 3 Results

### 3.1 PDPK1 expression was upregulated in EOC and was associated with poor prognosis

**Fig. 1:**
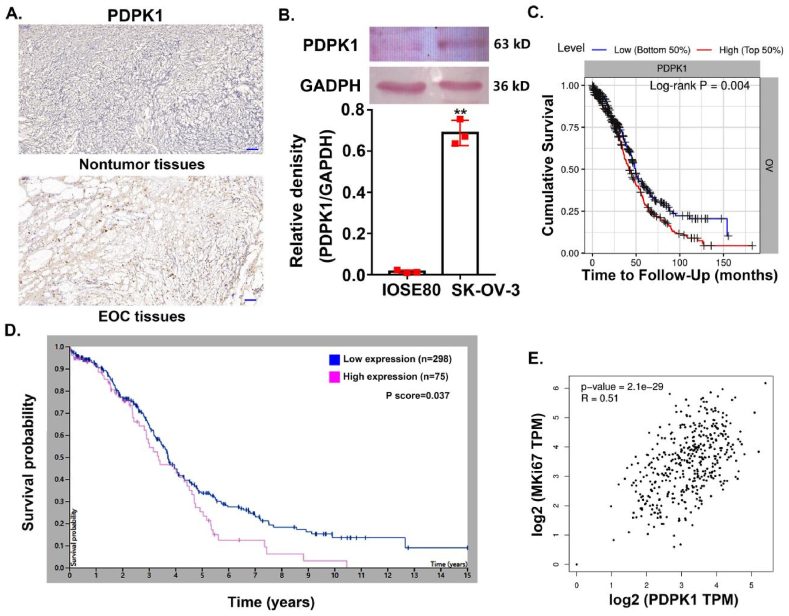
High expression of PDPK1 in EOC associated with poor prognosis. (A) IHC staining of EOC tissue and benign ovarian tissues. Scale bar = 100 μm. The magnification focus is 10×. (B) Western blotting analysis of PDPK1 expression in IOSE80 and SK-OV-3 cells, ∗∗*p* < 0.01, compared with IOSE80 cells. (C, D) The relationship between PDPK1 expression in ovarian cancer tissues and the prognosis of patients was analyzed based on TIMER and HPA databases. (E) The correlation between PDPK1 mRNA expression and MKi67 mRNA expression in ovarian cancer tissues was analyzed based on the GEPIA database.

For the identification of the role of PDPK1 in the process of EOC, the protein expression profiles of PDPK1 were examined in EOC tissues. As opposed to normal ovarian tissues, EOC tissues showed stronger positive IHC staining of PDPK1 in both the cytoplasm and nucleus (Fig. 1A). Further, western blotting analysis confirmed that PDPK1 expression was markedly increased in SK-OV-3 cells, compared with IOSE80 cells (Fig. 1B). Subsequently, we analyzed datasets from Tumor Immune Estimation Resource (TIMER, https://cistrome.shinyapps.io/timer/) and the Human Protein Atlas (HPA, https://www.proteinatlas.org/). We found a correlation between high PDPK1 expression and worse prognosis of ovarian cancer patients (Fig. 1C and D), which suggested that PDPK1 may play an oncogenic role in EOC. Moreover, we performed the data mining of the ovarian cancer cohort using the Gene Expression Profiling Interactive Analysis database (GEPIA, https://gepia.cancer-pku.cn/), which showed the mRNA expression levels of PDPK1 and MKi67 were positively correlated (Fig. 1E). In conclusion, these data demonstrated that PDPK1 expression was up-regulated in EOC and it was associated with poor prognosis.

### 3.2 E2 up-regulation of PDPK1 expression at the protein level via ESR1

**Fig. 2:**
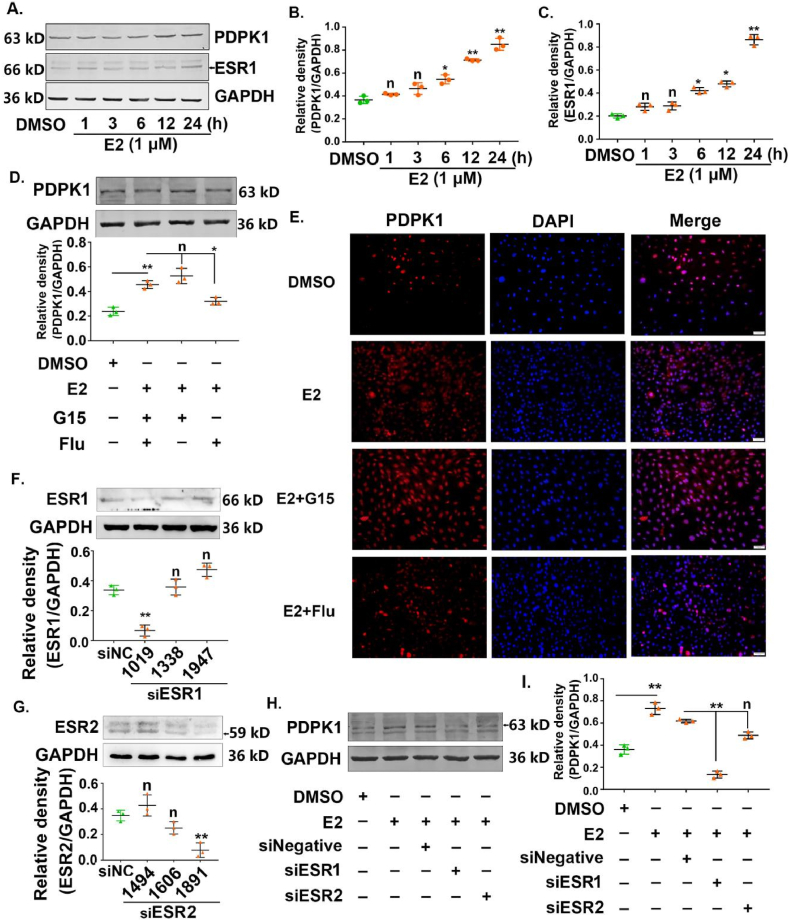
E2 increased PDPK1 expression at the protein level via ESR1. (A) Protein expression of ESR1 and PDPK1 in E2-induced SK-OV-3 cells. The cells were treated with 1 μM E2 at different times. (B, C) Statistical histogram of Fig. 2A. ∗*p* < 0.05, ∗∗*p* < 0.01, compared with the SK-OV-3 cells treated with DMSO. (D) Protein expression of PDPK1 in E2-induced SK-OV-3 cells under different treatments. ∗*p* < 0.05, ∗∗*p* < 0.01.(E) Representative images of immunofluorescence assay of PDPK1 protein expression in E2-induced SK-OV-3 cells treated with inhibitor. PDPK1 (red), DAPI (blue), the scale bar = 100 μm. (F) Protein expression of ESR1 in SK-OV-3 cells treated with siRNA-ESR1. ∗∗*p* < 0.01, compared with the SK-OV-3 cells treated with siRNA-Negative control. (G) Protein expression of ESR2 in SK-OV-3 cells treated with siRNA-ESR2. ∗∗*p* < 0.01, compared with the SK-OV-3 cells treated with siRNA-Negative control. (H) Protein expression of PDPK1 in SK-OV-3, siRNA-NC SK-OV-3, siRNA-ESR1 SK-OV-3, and siRNA-ESR1 SK-OV-3 cells treated with 1 μM E2. I. Statistical histogram of Fig. 2H. ∗∗*p* < 0.01.

According to our hypothesis, PDPK1 may be a downstream target of the E2 signaling pathway. In this regard, we detected the expression profiles of ESR1 and PDPK1 in E2-stimulated SK-OV-3 cells. We performed Western blotting, and our results demonstrate that E2 at a concentration of 1 μM up-regulated the protein expression levels of ESR1 and PDPK1 in a time-dependent manner (Fig. 2A). The up-regulation of ESR1 expression verified the stimulating effect of E2. Further, the function of E2 significantly increased the ESR1 and PDPK1 expression after 6 h of stimulation, as depicted in Fig. 2B and C. It is known that the estrogen signaling pathways are divided into genomic and non-genomic. While the genomic pathway depends more on the transcriptional regulation of target genes through transcription factor ERs, the non-genomic pathway partially mediates the rapid activation of the signal cascade through the membrane-bound G protein-coupled estrogen receptor (GPR30). To exclude E2 affected the expression of PDPK1 through non-genomic pathway, we observed the expression level of PDPK1 in E2-induced SK-OV-3 cells under the GPR30 selective antagonist G15 or the treatment of ER inhibitor Fulvestrant. We observed that Fulvestrant co-treatment offset the effect of E2 on the protein levels of PDPK1; however, the G15 co-treatment did not change the E2-induced effects on the protein levels of PDPK1 (Fig. 2D). Furthermore, immunofluorescence experiments also confirmed that Fulvestrant affected the expression of PDPK1 in E2-induced SK-OV-3 cells (Fig. 2E).

To validate and further identify the functional relationship between E2 and PDPK1 expression in SK-OV-3 cells, ESR1 or ESR2 was knocked down in SK-OV-3 cells, and the interference efficiency of related siRNA was evaluated using western blotting (Fig. 2F and G). Accordingly, we observed that as compared with the negative siRNA transfection group, ESR1/ESR2 siRNA explicitly blocked the expression of ESR1/ESR2. After the siRNA-mediated knockdown of ESR1-1019, the PDPK1 expression levels decreased; however, the ESR2-homo-1891 siRNA did not affect the expression of PDPK1. (Fig. 2H and I). These results suggested that the regulation of PDPK1 expression via E2 at the protein level occurs through ESR1.

### 3.3 The depletion of PDPK1 suppressed E2-driven SK-OV-3 cells proliferation and migration but promoted apoptosis

**Fig. 3:**
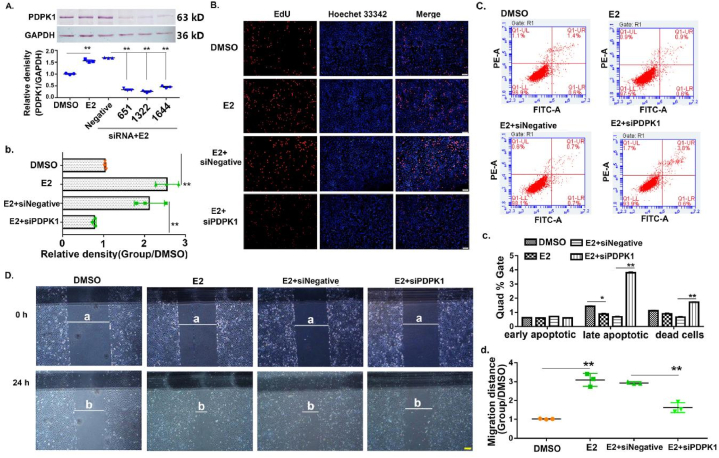
Knock-down of PDPK1 suppresses E2-driven SK-OV-3 cells proliferation and migration but promotes apoptosis. (A) Protein expression of PDPK1 in SK-OV-3 cells treated with siRNA-PDPK1. ∗∗*p* < 0.01, compared with the SK-OV-3 cells treated with siRNA-Negative control. (B) Representative images of SK-OV-3 cells' proliferation ability under different treatments were detected by EdU staining. EdU‐staining SK-OV-3 cells (red), Hoechst 33342 staining nucleus (blue), the scale bar = 100 μm. b. Statistical histogram of Fig. 3B. ∗∗*p* < 0.01. (C) Representative images of SK-OV-3 cells' migration ability under different treatments were detected by wound healing assay. The scale bar = 200 μm. The magnification focus is 4×. c. Statistical histogram of Fig. 3C. ∗∗*p* < 0.01. (D) Representative images of SK-OV-3 cells undergoing apoptosis under different treatments as detected by flow cytometry. PI‐A (late apoptotic cells), AV‐FITC‐V (early apoptotic cells). (E) Statistical histogram of Fig. 3D. ∗*p* < 0.05, ∗∗*p* < 0.01.

To elucidate the effect of PDPK1 in E2-driven SK-OV-3 cells, we examined the physiological functions of SK-OV-3 cells with PDPK1 knockdown under E2 stimulation, including changes in cells proliferation, migration, and apoptosis levels. Initially, we identified the most effective siRNA to block PDPK1 protein expression using western blotting analysis. Fig. 3A showed that PDPK1-homo-1322 siRNA was the most effective in preventing the protein expression of PDPK1. Further, EdU staining was used to detect the effect of PDPK1 on the proliferation of SK-OV-3 cells stimulated by E2. Furthermore, it was observed that E2 demonstrated a notable capacity to facilitate the proliferation of SK-OV-3 (Fig. 3B and b), which was in accordance with the preceding conclusion [30]. In contrast, the proliferative effect of E2 on SK-OV-3 cells was significantly inhibited by PDPK1 knockdown. Subsequently, the cell migration assay demonstrated that E2 strongly promoted the migration of SK-OV-3 cells; however, the migration potency of E2 was weakened by the depletion of PDPK1, as shown in Fig. 3C and c. Since the aforementioned results indicated that the depletion of PDPK1 suppressed E2-driven SK-OV-3 cell proliferation and migration, we investigated the potential roles of E2 and PDPK1 in altering the apoptosis of SK-OV-3 cells by performing flow cytometry. We noted that E2 induced apoptosis later (Fig. 3D and d), and the interference with PDPK1 aggravated late apoptosis and the death of cells. In conclusion, these results illustrated that E2 showed a marked cancer-promoting effect in SK-OV-3 cells in a PDPK1-dependent manner.

### 3.4 Overexpression of PDPK1 was positively correlated with the potency of E2 to promote EOC proliferation and metastasis in vitro

**Fig. 4:**
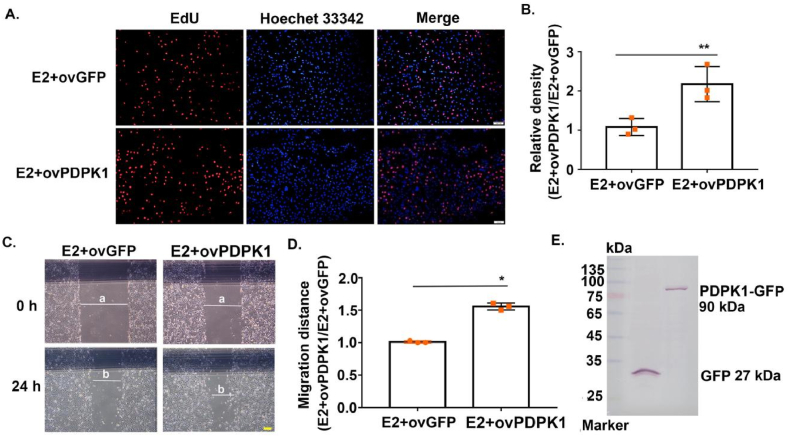
Overexpression of PDPK1 promotes E2-induced proliferation and metastasis in SK-OV-3 cells. (A) Representative images of EdU staining to detect the proliferation ability of SK-OV-3 cells overexpressing PDPK1 under E2 treatment as compared with SK-OV-3 cells overexpressing GFP. EdU‐staining SK-OV-3 cells (red), Hoechst 33342 staining nucleus (blue), scale bar = 100 μm. (B) Statistical histogram of Fig. 4A, ∗∗p < 0.01, compared with the negative control. (C) Representative images of wound healing assay to detect the migration ability of SK-OV-3 cells overexpressing PDPK1 under E2 treatment, compared with SK-OV-3 cells overexpressing GFP. Scale bar = 200 μm. The magnification focus is 4×. (D) Statistical histogram of Fig. 4C. ∗*p* < 0.05, compared with the negative control. (E) Protein expression of PDPK1 in SK-OV-3 cells overexpressing GFP and SK-OV-3 cells overexpressing PDPK1.

To further prove whether the carcinogenic effect of E2 was PDPK1-dependent, we constructed SK-OV-3 cells that overexpressed PDPK1 for our subsequent experiments. The overexpression of PDPK1 significantly enhanced the EdU signal in E2-induced SK-OV-3 cells as compared to the overexpression of GFP (Fig. 4A and B). Similarly, wound healing assay showed that SK-OV-3 cells in the PDPK1 overexpression group showed a stronger distance of migration (Fig. 4C and D). Further, the successful construction of transiently transfected cell lines is demonstrated in Fig. 4E. The results herein indicated that PDPK1 promoted E2-induced cell proliferation and migration of SK-OV-3 cells.

### 3.5 E2 regulated relative genes expression through PDPK1 to determine cell fate

**Fig. 5:**
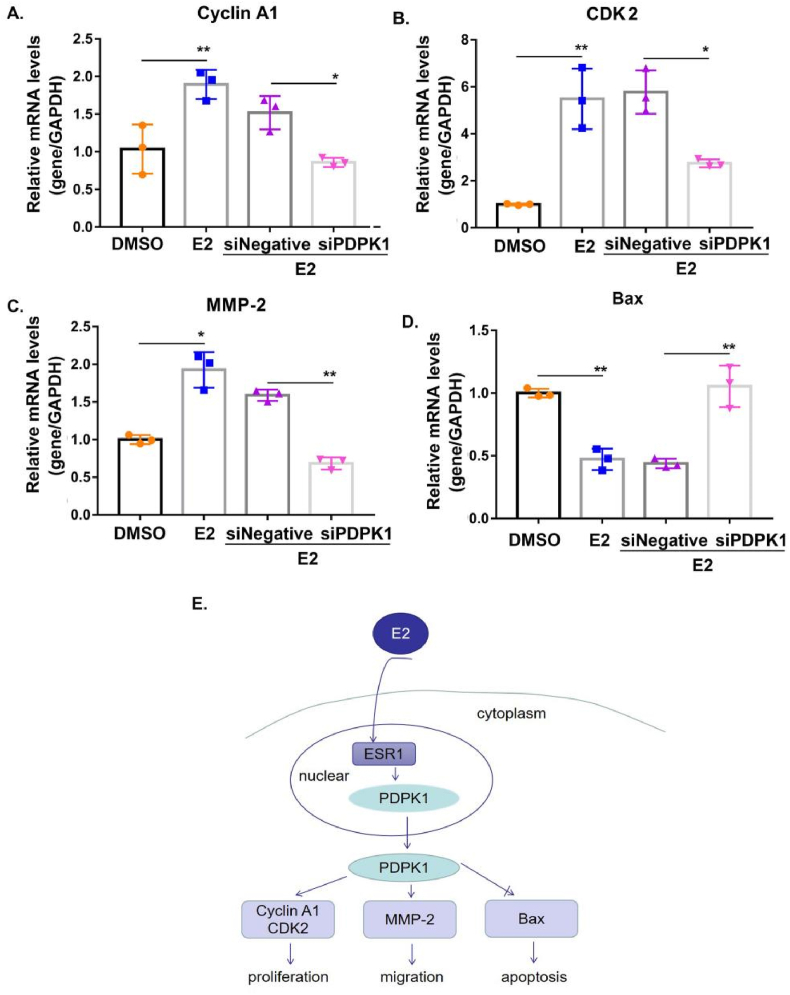
E2 regulates relative genes expression through PDPK1 to determine cell fate. (A–D) The mRNA expression levels of Cyclin A1 (A), CDK2 (B), MMP-2 (C), and Bax (D) in E2-induced SK-OV-3 cells after PDPK1 knock down. ∗*p* < 0.05, ∗∗*p* < 0.01, compared with the negative control. (E) Proposed signaling pathways involved in E2-induced PDPK1 overexpression and E2 promotes cell proliferation and metastasis in SK-OV-3 cells but promotes apoptosis via PDPK1. E2 application increases PDPK1 protein expression level through ESR1, and an increase in PDPK1 induces relative genes expression.

To clarify the mechanism of E2 regulation of PDPK1 on SK-OV-3 cell fate, we examined the mRNA expression levels of the proliferation genes Cyclin A1 and CDK2, migration relative gene MMP-2, and the apoptosis relative gene Bax. The mRNA expression levels of Cyclin A1, CDK2, and MMP-2 increased significantly under E2 stimulation; however, the interference of PDPK1 expression prevented this trend (Fig. 5A–C). Notably, the mRNA expression level of Bax in DMSO-induced SK-OV-3 cells was significantly higher than that in the E2 treatment group. Further, after the knockout of PDPK1, the expression of Bax in E2-induced SK-OV-3 cells increased as compared to the control group (Fig. 5D). Thereby, we demonstrated that E2 regulated the mRNA expression of related genes via PDPK1, which further verified that PDPK1 is involved in E2-mediated effects on the biological functions of EOC (Fig. 5E).

## 4 Discussion

PDPK1 is a member of the AGC kinase family and it consists of two domains, namely, the N-terminal kinase domain and the C-terminal PH domain [13]. Many studies now corroborate the conclusion that PDPK1 contributes to the progression of cancers and shows high expression in cancers, such as breast, gastric, and prostate cancer. Interestingly, our data also manifested the high expression of PDPK1 in EOC tissues and SK-OV-3 cell. Moreover, combined with bioinformatics analyses, we found that the high expression of PDPK1 is closely related to the poor prognosis of patients with ovarian cancer, which implies that PDPK1 can impact the development of ovarian cancer. Recently, PDPK1 has been reported to be closely related to the invasion of ovarian cancer. However, the molecular mechanism of the involvement of PDPK1 in the development of EOC remains indistinct.

EOC is the most lethal malignant estrogen-sensitive gynecological cancer―its 5-year survival rate post-diagnosis is approximately 45 % and represents the seventh most commonly diagnosed cancer worldwide, which accounts for 90 % of ovarian cancers [33]. However, there is scarce evidence regarding the molecular mechanism of its pathogenesis. EOC is considered to be regulated by hormones, and an abnormal level of estrogen drives its growth [9]. Although estrogen plays an important role in the development of EOC, the molecular mechanism of how estrogen mediates tumorigenesis remains unclear. And it is worth noting that there is no evidence regarding the potential mechanisms of estrogen-induced PDPK1 in the progression of EOC. In our current study, the data showed that the effects of estrogen and PDPK1 expression in SK-OV-3 cells had a positive correlation, and that estrogen upregulated PDPK1 expression in a time-dependent manner. The function of estrogen is mediated by two different signaling pathways, including genomic and non-genomic signaling pathways. The biological functions of estrogen are not only reflected in the regulation of downstream target gene expression through the nuclear receptor ERs in the genomic pathway, but also through binding to its membrane receptors (GPR30), triggering downstream protein modifications and translocation in the non-genomic pathway [34,35]. To further identify the signaling pathway whereby estrogen upregulated the expression of PDPK1, a co-culture of G15 or fulvestrant with estrogen was performed to confirm that estrogen regulated the expression of PDPK1 through its nuclear receptor ERs. It is known that ERs, including ESR1 and ESR2, bind to estrogenic response elements (EREs) in the gene promoter regions for participation as transcription factors in the transcription of downstream target genes [36]. Our data indicate that ESR1, rather than ESR2, was involved in the upregulation of PDPK1 expression via genomic signaling pathways activation. This represents a new regulatory mechanism for EOC driven by estrogen.This result triggers our hypothesis that the effects of estrogen on the EOC cells were related to PDPK1. To prove this conjecture, we transformed SK-OV-3 cells with plasmids or siRNA to create model cells with diverse expressions of PDPK1. Our results demonstrated that diminished PDPK1 expression inhibited the proliferation and migration in estrogen-induced SK-OV-3 cells; however, they promoted apoptosis. On the contrary, the overexpression of PDPK1 increased the effects of estrogen on the viability and migratory ability of SK-OV-3 cells. Additionally, the knockdown of PDPK1 inhibited the mRNA expression of proliferation and migration-related genes (Cyclin A1, CDK-2, and MMP2) in SK-OV-3 cells induced by estrogen but promoted the expression of apoptosis-related genes (Bax) (Fig. 5). These results demonstrated that PDPK1 is an oncogene that is required for the estrogen-induced development of EOC.

PDPK1 plays a role in cancer-promoting in EOC, and it can be used as a therapeutic target for EOC. Our study also confirmed that estrogen can promote EOC development by upregulating the expression levels of PDPK1 via ESR1. However, this study had some limitations. Although we found that estrogen upregulated the expression of PDPK1 in vitro, the mechanism of regulation of PDPK1 via estrogen in vivo needs to be verified further, such as genetic mutations, epigenetic modifications, or environmental exposures. Secondly, the current experiments only verified the upstream regulatory mechanism of PDPK1 in EOC driven by estrogen. Hence, the downstream signaling pathway of PDPK1 in EOC driven by estrogen warrants further exploration.

## 5 Conclusion

We reported novel findings regarding the high expression levels of PDPK1 in EOC tissues and SK-OV-3 cells. Further, the high PDPK1 expression level was closely related to the poor prognosis of patients with ovarian cancer. We used SK-OV-3 cells as a model and determined that estrogen promoted EOC cells proliferation and migration but inhibited apoptosis by increasing the expression of PDPK1 via ESR1. The aforementioned results elucidate new evidence regarding the mechanism by which PDPK1 promotes EOC driven by estrogen. This implies that PDPK1 development targeted inhibitors that may benefit the treatment of patients with EOC.

## CRediT authorship contribution statement

**Yajie Wang:** Writing – original draft, Methodology. **Huanchao Chang:** Methodology, Investigation, Conceptualization. **Xiuwen Li:** Software, Methodology, Investigation. **Hairong Zhang:** Formal analysis, Data curation. **Qianqian Zhou:** Resources. **Shengjian Tang:** Writing – review & editing, Supervision. **Di Wang:** Writing – review & editing, Supervision, Funding acquisition.

## Ethics approval and consent to participate

This study was approved by the Ethics Committee on Human Research of the Shandong Second Medical University (ethics approval number: 063/2018; ethics approval date: 2018-2-22), and all donors provided written informed consent.

## Consent for publication

All authors have approved the manuscript for submission.

## Data availability statement

Data included in article/supplementary material/referenced in article.

## Funding statement

This work was supported by grants from the 10.13039/501100001809National Natural Science Foundation of China [Grant number: 81802616] and Weifang science and technology development plan [Grant number: 2024YX034].

## Declaration of competing interest

The authors declare that they have no known competing financial interests or personal relationships that could have appeared to influence the work reported in this paper.
