# Supplementary material for: Estrogen regulates PDPK1 to promote cell proliferation in epithelial ovarian cancer
Source: Heliyon. 2024 Nov 8;10(22):e40296. doi: 10.1016/j.heliyon.2024.e40296 (PMC11609661; doi:10.1016/j.heliyon.2024.e40296)
Supplement: Multimedia component 1 [file mmc1.doc]

**Supplementary**

**Estrogen regulates PDPK1 to promote cell proliferation in epithelial ovarian cancer**

Yajie Wang^1,2#^, Huanchao Chang ^2,3#^, Xiuwen Li^1,2#^, Hairong Zhang^2^, Qianqian Zhou^3^, Shengjian Tang^2*^, Di Wang^1,2*^

^1^School of Basic Medical Sciences, Weifang Medical University, Weifang, Shandong 261053, P.R. China.

^2^Plastic Surgery Institute, Weifang Medical University, Weifang, Shandong 261053, P.R. China.

^3^Affiliated Hospital of Weifang Medical University, Weifang, Shandong, China

^#^These authors contributed equally to this work.

^*^To whom correspondence should be addressed: Plastic Surgery Institute, Weifang Medical University, Weifang, Shandong 261053, P.R. China; E-mail: wang234di@163.com (Di Wang), tsj3676@163.com (Shengjian Tang) .

(A)


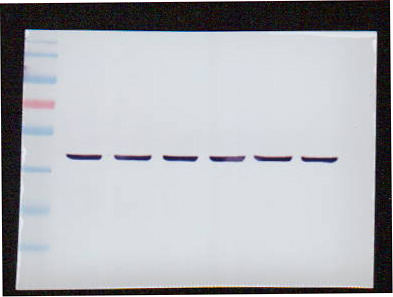


GAPDH


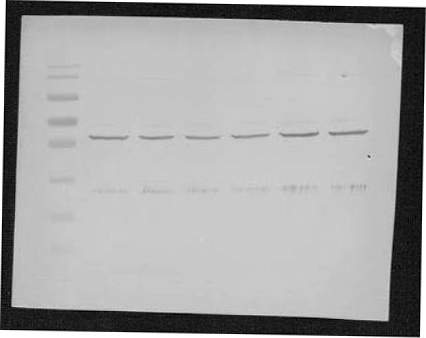


PDPK1


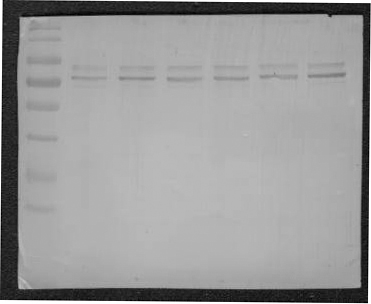


ESR1

(B)

PDPK1


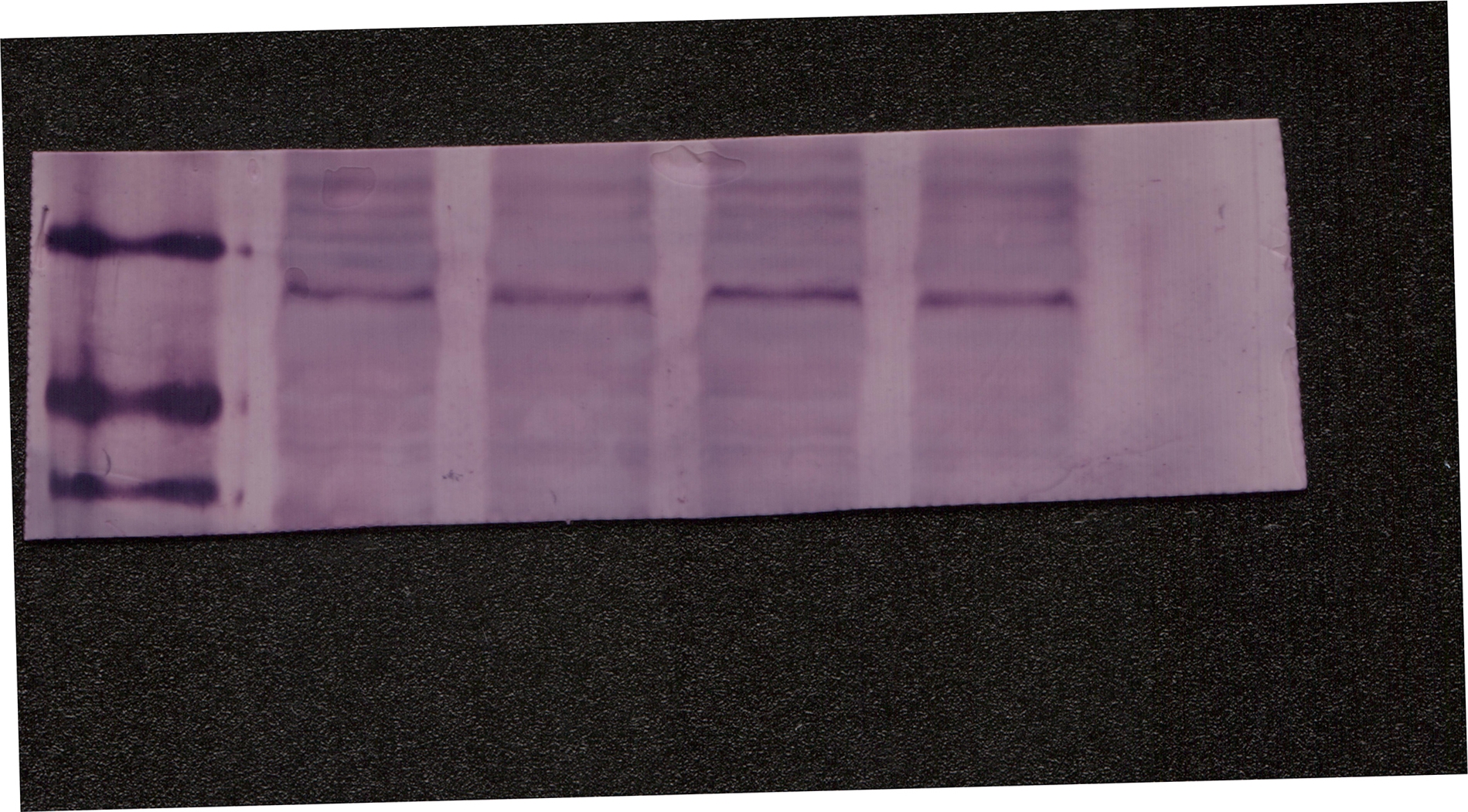

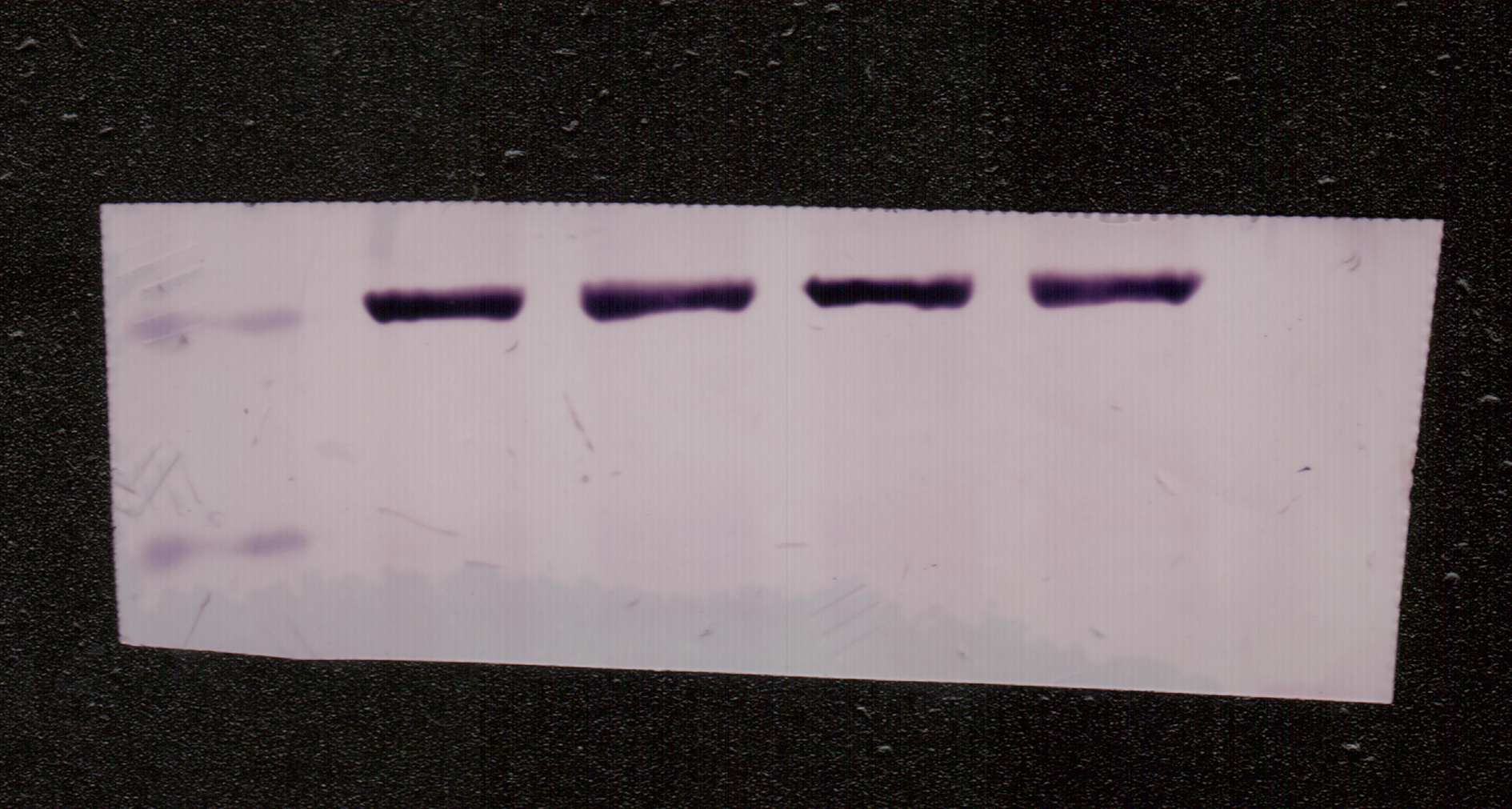


GAPDH

GAPDH


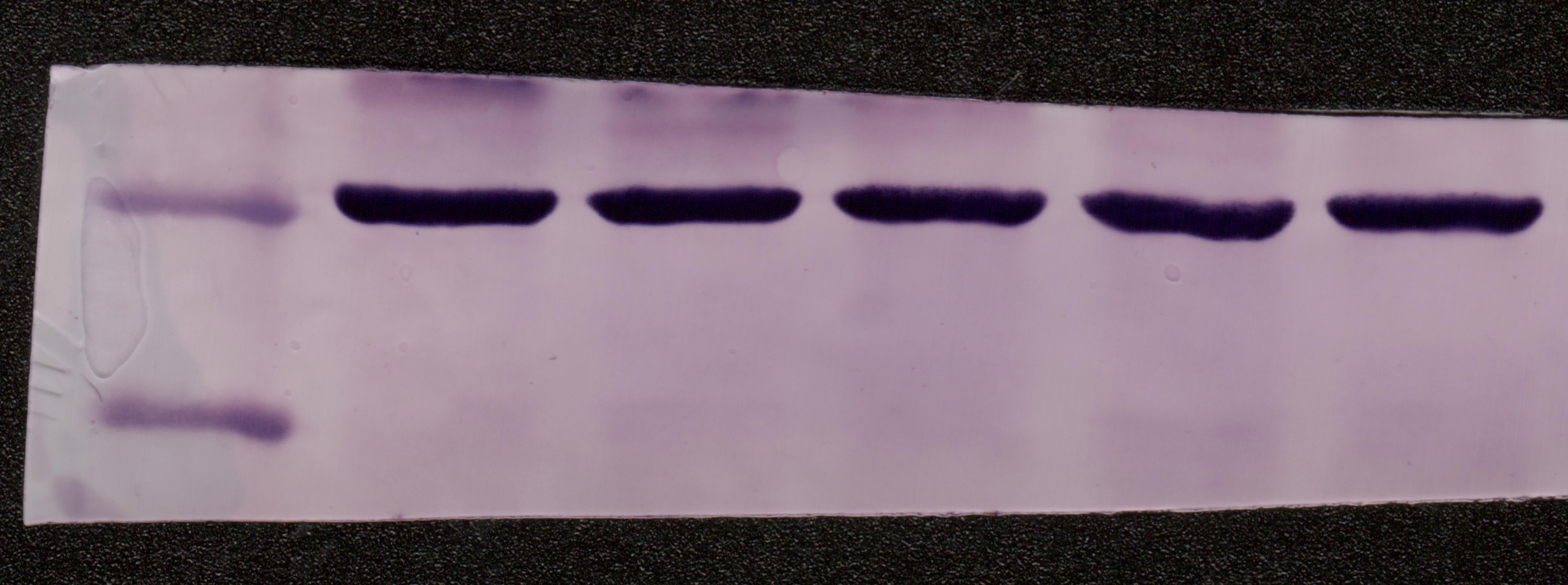


(E)

PDPK1


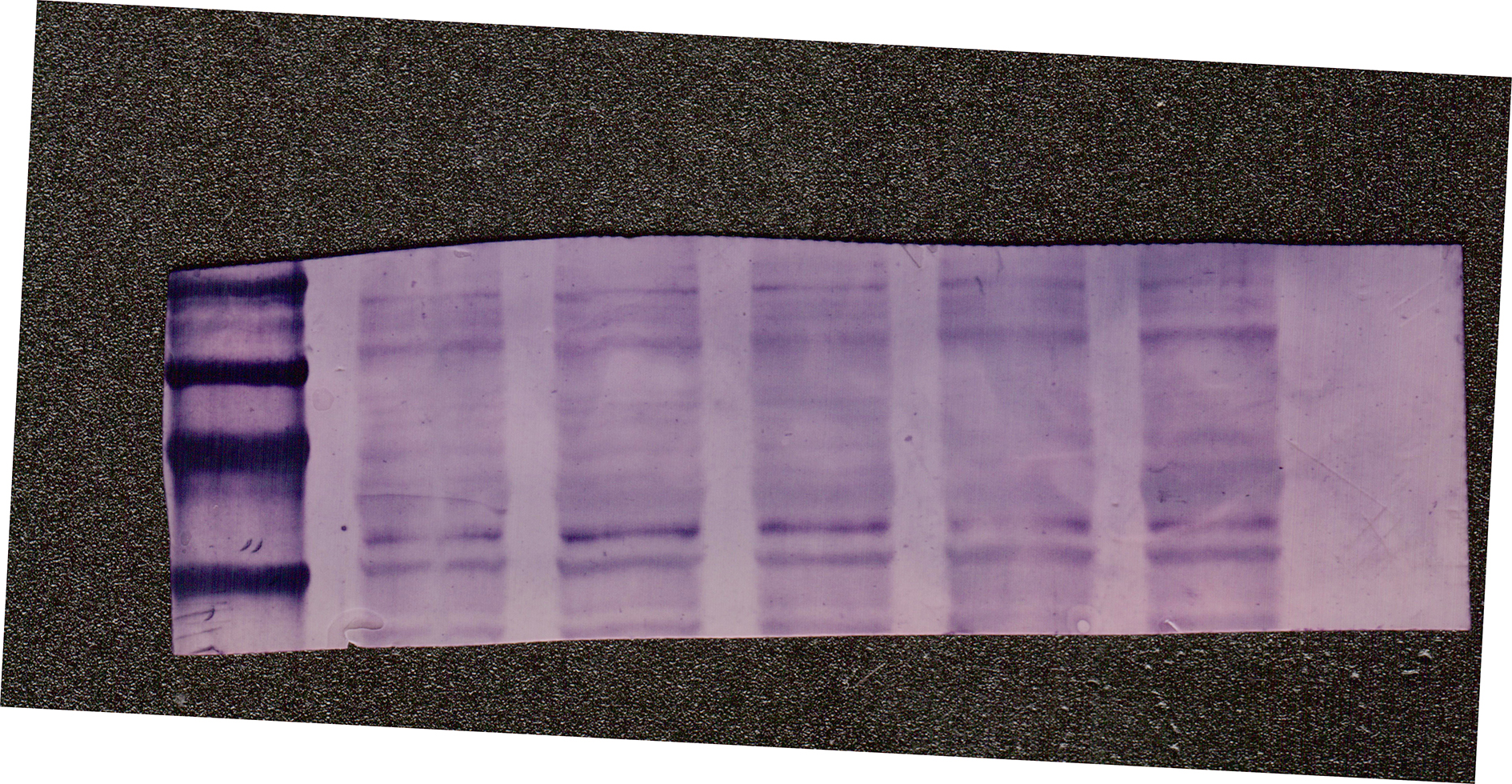


(F)

GAPDH

PDPK1


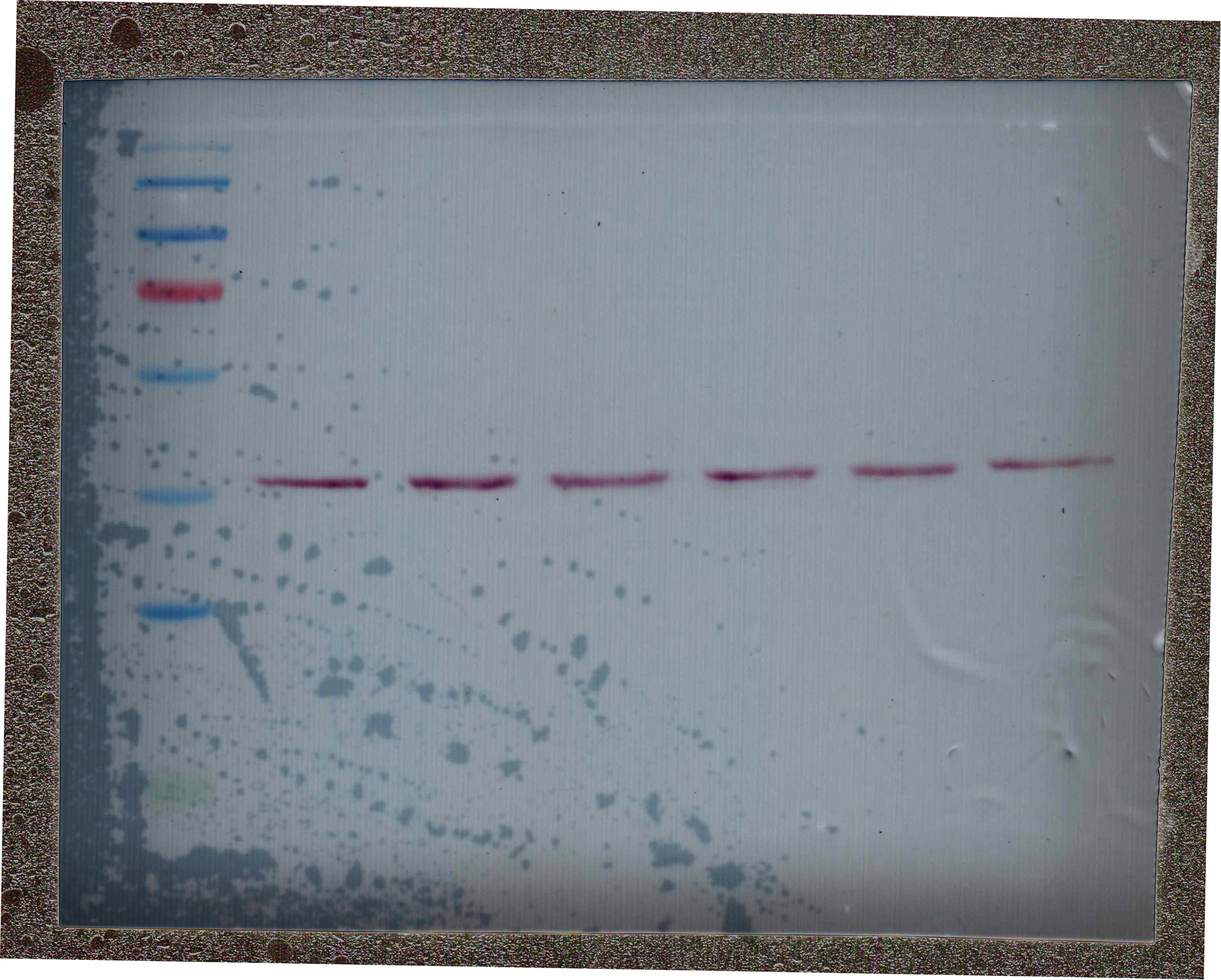

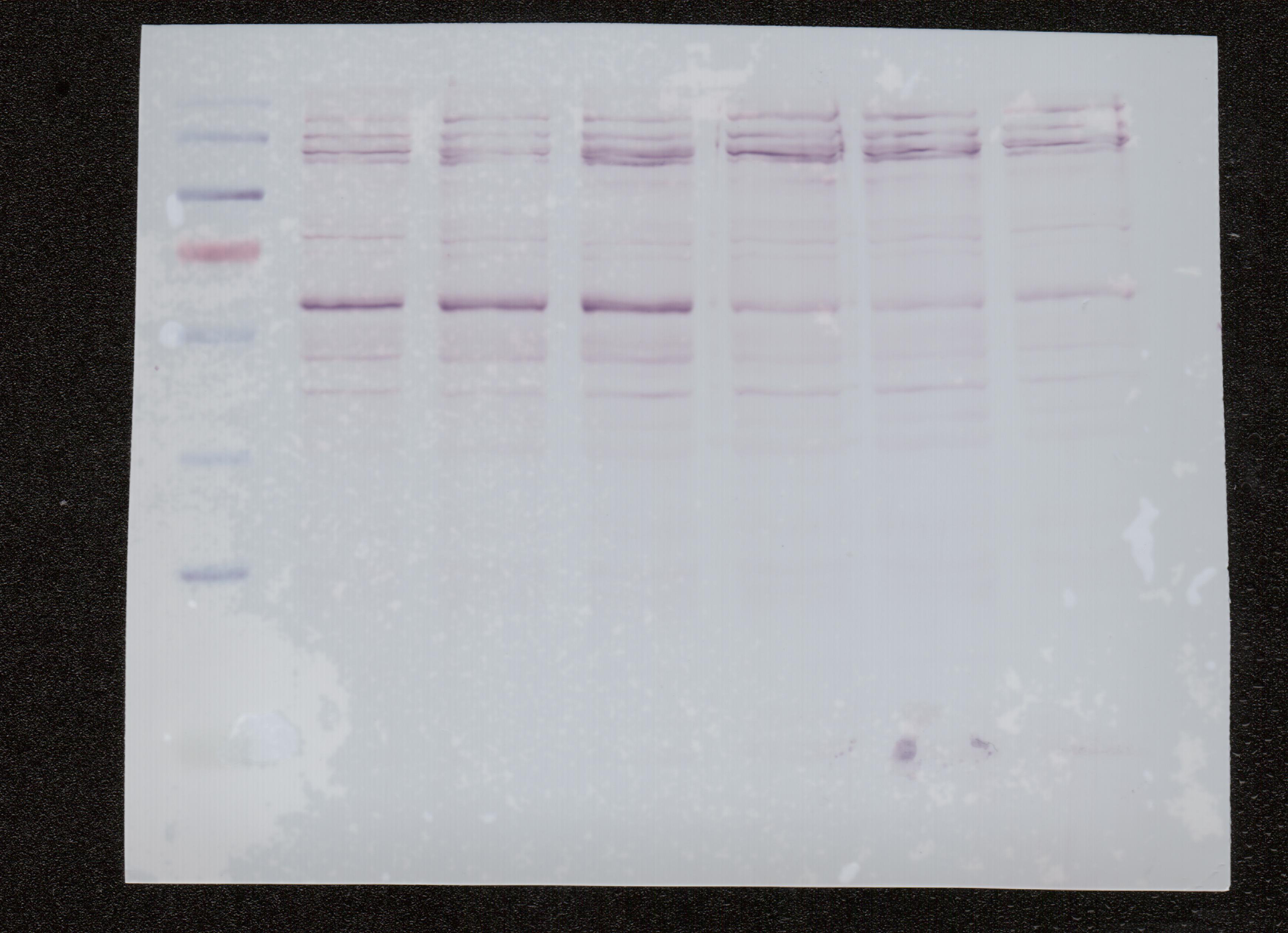


(G)


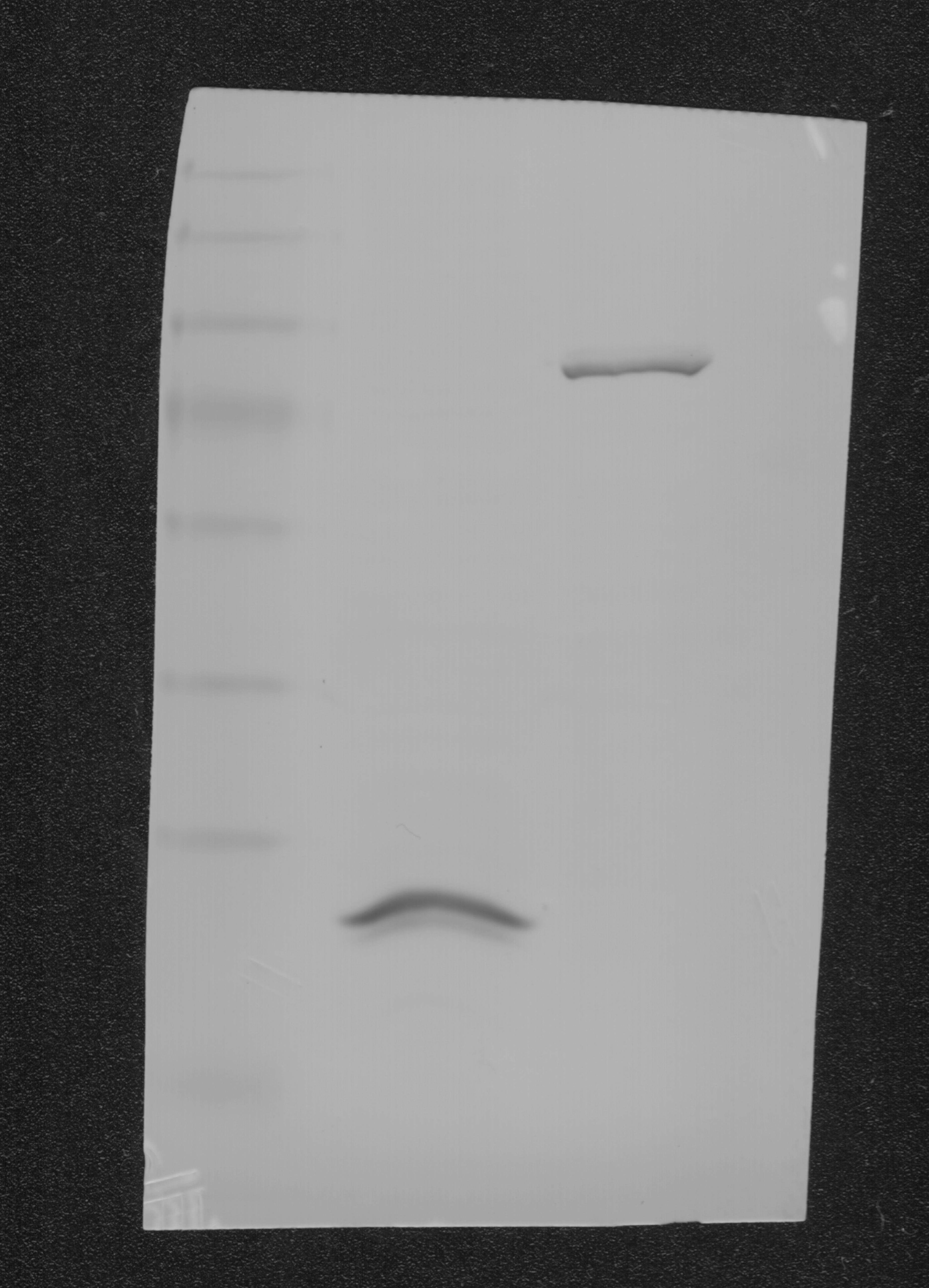


PDPK1-GFP

GFP

(C)

ESR1

GAPDH


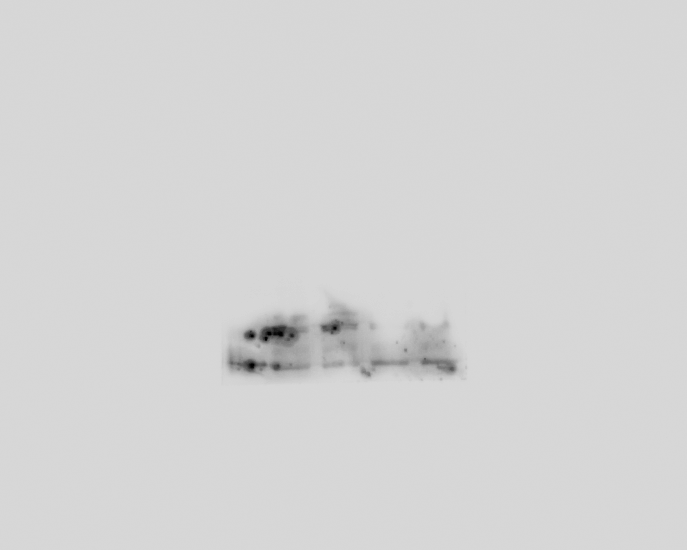

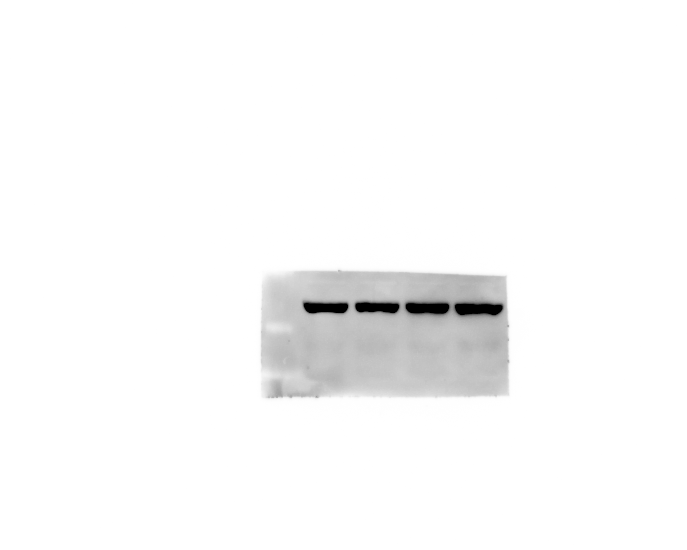


(D)

GAPDH

ESR2


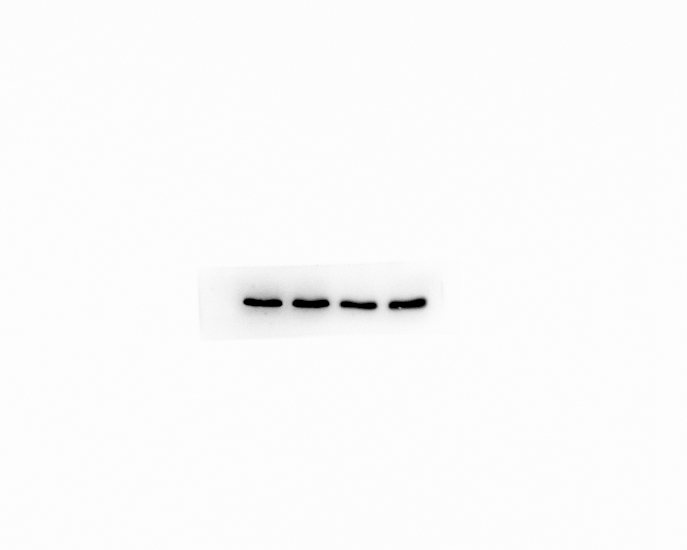

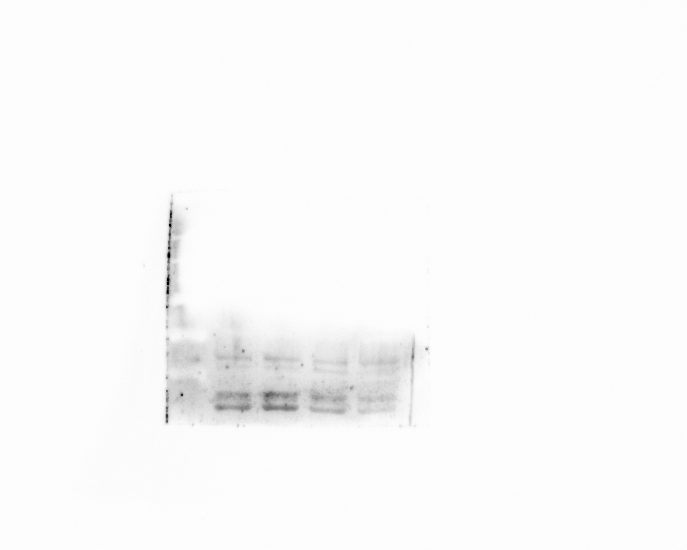


**Figure S1.** **Western blot figures.**(A) Uncropped and unadjusted versions of Figure 2A. (B) Uncropped and unadjusted versions of Figure 2D. (C) Uncropped and unadjusted versions of Figure 2F. (D) Uncropped and unadjusted versions of Figure 2G. (E) Uncropped and unadjusted versions of Figure 2H. (F) Uncropped and unadjusted versions of Figure 3A. (G) Uncropped and unadjusted versions of Figure 4E.
